# Candida laryngitis appearing as carcinoma 

**Published:** 2015

**Authors:** Keivan Kiakojuri, Mehdi Dehghan, Mohammad Reza Hasanjani Roushan, Bijan Pourdahash

**Affiliations:** 1 Department of Otolaryngology, Babol University of Medical Sciences, Babol, Iran.; 2 Department of Speech Therapy, Babol University of Medical Sciences, Babol, Iran.; 3 Infectious Diseases and Tropical Medicine Research Center, Babol University of Medical Sciences, Babol, Iran.; 4 Babol Pathology Center, Babol, Iran.

**Keywords:** Laryngeal Candidiasis, Hoarseness

## Abstract

**Background:**

Focal laryngeal candidiasis is not reported widely and is very infrequently recognized clinically. This disease is rare and may occur after pulmonary, pharyngeal and esophageal candidiasis or as part of disseminated disease. It is also secondary to inhaled steroid therapy which is usually mild and has been reported in 10-15 percent of patients taking these medications.

**Case Presentation:**

In this study, we introduced a rare case of laryngeal candidiasis in a 79-year-old immunocompromised male presented with 17 months of progressive hoarseness. In video laryngoscopy a white, vegetative mass on anterior one-third of right vocal cord mimicking laryngeal carcinoma. The histopathological examination showed laryngeal mucosal with keratosis, degenerating necrotic epithelial cell aggregates containing hyphea and candida albicans.

**Conclusion:**

In immunocompromised patients, the diagnosis of laryngeal candidiasis should be considered in any patients with laryngeal symptoms

Respiratory fungal infections should be considered in immunocompromised patients who have received either long-term steroid treatment, broad-spectrum anti-microbial therapy or have a non-resolving underlying chronic disease. In other words, respiratory fungal infections are relatively prevalent conditions (1-3). However, laryngeal fungal infections can be presented in various diseases such as gastroesophageal reflux disease, granulomatous disease, leukoplakia and carcinoma (4-8) which mislead the treating team to get correct diagnosis and management. Therefore, identifying the lesion at the earliest stage of disease is very important to avoid morbid or life-threatening consequences (6). Candida albicans is an organism that normally makes a quiet home for itself on host’s skin and does not bother anyone. We all carry this organism on our skin, in our mouth, in our gastrointestinal tract and in the case of women, in the vagina. Occasionally the yeast multiplies uncontrollably causing pain and inflammation. By far, the most common problems are skin, mouth and vaginal infections, it may also infect the internal organs (9). But laryngeal candidiasis is not recognized widely or may be clinically documented and reported infrequently (1). This disease may occur after pulmonary, pharyngeal and esophageal candidiasis or as part of disseminated diseases (10, 11). It is also secondary to inhaled steroid therapy which is usually mild and has been reported in 10-15 percent of patients complaining dysphonia during treatment (3, 12). In the largest case series study by Wong et al. in 2009, only 54 patients were reported during a 10 – study period from 1995 to 2005 (13).

Few cases were reported previously. We report a case of laryngeal candidiasis with an unusual mimicking laryngeal carcinoma to describe the clinical and histological features of this condition and highlight the role of early diagnosis and treatment.

## Case Reports

A 79-year – old man presented to the E.N.T clinic at Ayatollah Rouhani Hospital of Babol, Iran, with 17 months of progressive hoarseness. He was panegyrist whose voice was getting worse during the eulogy. We referred him to speech therapy clinic video laryngoscopy. During video laryngoscopy, a white, vegetative mass on anterior one-third of right vocal cord was shown. 

**Figure 1:**
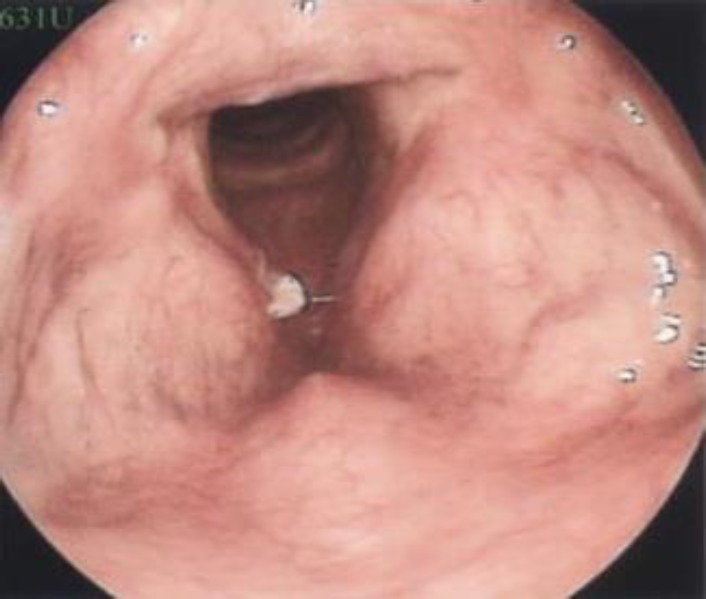
Video laryngoscopy of vocal cord

At first glance, it mimicked laryngeal carcinoma, so the decision for surgery was made by the surgeons. Under anesthesia, biopsy of the lesion was carried out to rule out malignancy. Histopathological examination revealed laryngeal mucosal with keratosis, degenerating necrotic epithelial cell aggregates containing hyphea and Candida albicans.

**Figure 2:**
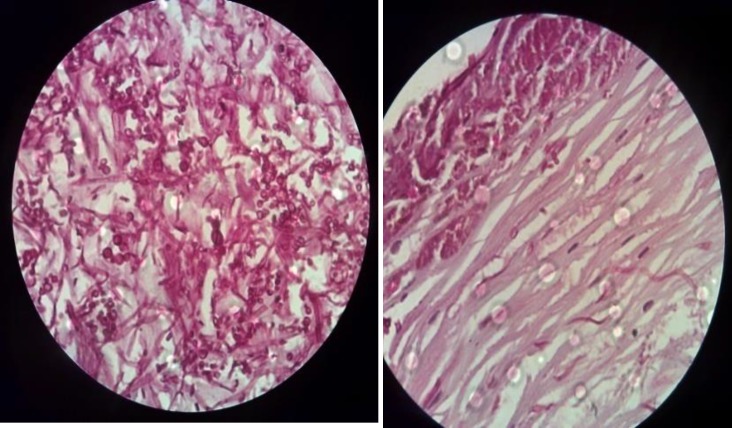
Parthology of vocal cord

The patient was treated with 200 mg of oral itraconazole once daily for 2 weeks. Video laryngoscopy after 2 weeks showed an improvement of the right vocal cord on the affected area. 

**Figure 3:**
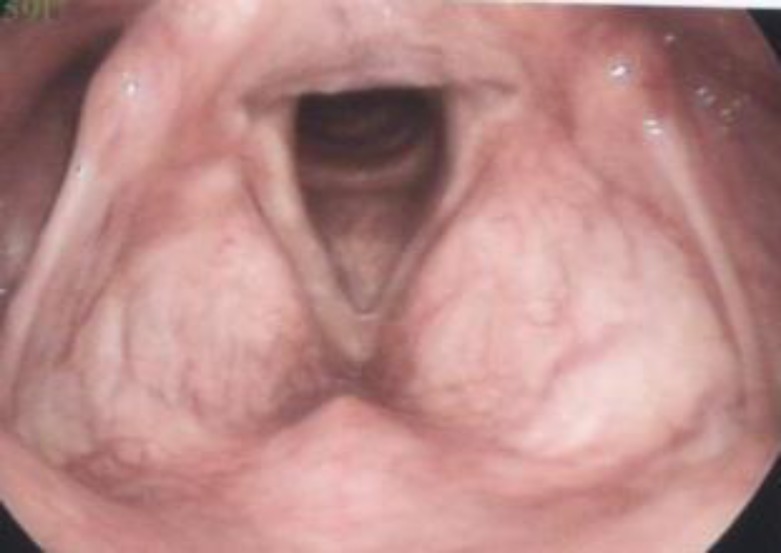
Video laryngoscopy of vocal cord after treatment

## Discussion

Laryngeal fungal infections must be considered in the different diagnosis of chronic laryngitis (1). A number of factors can increase the chance of the yeast growing out of control. The leading cause is the overuse of antibiotics. When we take antibiotics to deal with less friendly bacteria, we kill some harmless ones as well. Yeast which is unaffected by antibiotics moves in to the vacated spots once occupied by bacteria and starts to grow and multiply (1, 9). Steroids and some cancer medications weaken the immune system and can allow yeast to flourish. A year infection of the mouth (known as oral trash) most often develop in people with disease such as cancer and AIDS (1, 9, 14).

They can also develop in people with diabetes or in people who have long – term irritation resulting from dentures. Laryngeal candidiasis always expands from oral cavity or due to oral trash and it may result in the involvement of esophagus (5, 9, 15). The diagnosis of laryngeal candidiasis is suggested when white, curd – like patches are seen in the larynx but it can mimic laryngopharyngeal reflux disease (LPR), granulomatous disease, leukoplakia and carcinoma (4, 8, 11).

The most common symptom of laryngeal candidiasis is hoarseness, and dysphagia however, stridor and respiratory distress are reported infrequently (11, 16). A non–invasive method to diagnose laryngeal disease is video laryngoscopy which typically reveals hyperplasia, oedema, white curd like patches and white or grey pseudoepitheliomatosis in patients with candidiasis (1, 11, 17). Since vocalization of the vocal cords need to vibration of epithelial layer and making mucosal waves, so conceding to integration of mucosal waves is very important because candidiasis affects superficial layer of the vocal cords. In this way strobovideolaryngoscopy provides better information of mucosal waves (11, 16, 18). Marked stiffness and interference of mucosal waves may occur on stroboscopy, 18). Biopsy is not most commonly used in candidiasis, however, if there is probability of malignancy (as in this case) or any other serious systematic disease, or if there is incomplete response to adequate therapy, the use of biopsy will be acceptable. Biopsy represents pseudoepitheliomatosis hyperplasia with yeast forms and pseudohyphe. On histopathological examination, a fungal infection can be confirmed (5, 8, 19). All patients described in the literature have responded to conservative treatment including various anti-fungal drugs from intravenous amphotericin to oral flunconazole or ketoconazole to topical nystatin based on the severity of the disease. The duration of treatment varying from one week to one month depends on the scope of clinical progression (12, 14, 20). To evaluate the improvement or recurrence of disease, video laryngoscopy is suggested (6, 10). In conclusion, early diagnosis and treatment of candidiasis are important to prevent the spread of infection. Insufficient biopsy or inadequate treatment can lead to vocal function deficit especially on making integration of mucosal waves in vocalization time. Nevertheless, fungal infection can cover an underlying tumor progression, so biopsy and histopathological evaluation are necessary to early diagnose and rule out possible malignancy.
